# Can Artificial Intelligence Improve the Energy Efficiency of Manufacturing Companies? Evidence from China

**DOI:** 10.3390/ijerph19042091

**Published:** 2022-02-13

**Authors:** Jun Liu, Yu Qian, Yuanjun Yang, Zhidan Yang

**Affiliations:** 1 School of Management Science and Engineering, Nanjing University of Information Science & Technology, Nanjing 210044, China; qianyu7098@gmail.com (Y.Q.); yyjwork1995@163.com (Y.Y.); hannahyeung1226@gmail.com (Z.Y.); 2 Institute of Free Trade Zone, Nanjing University of Information Science & Technology, Nanjing 210044, China

**Keywords:** artificial intelligence, manufacturing enterprises, energy efficiency, heterogeneity

## Abstract

Improving energy efficiency is an important way to achieve low-carbon economic development, a common goal of most nations. Based on the comprehensive survey data of enterprises above a designated size in Guangdong Province, this paper studies the impact of artificial intelligence on the energy efficiency of manufacturing enterprises. The results show that: (1) artificial intelligence, as measured by the use of industrial robots, has significantly improved the energy efficiency of manufacturing enterprises. This conclusion is still robust after introducing data on industrial robots in the United States over the same time period as the instrumental variable for the endogeneity test. (2) The mechanism test shows that artificial intelligence mainly promotes the improvement in energy efficiency by promoting technological progress; the impact of artificial intelligence on the technological efficiency of enterprises is not significant. (3) Heterogeneity analysis shows that the age of the manufacturing enterprises inhibits a promoting effect of artificial intelligence on energy efficiency; manufacturing enterprises’ performance can enhance the promoting effect of artificial intelligence on energy efficiency, but this promoting effect can only be shown when the enterprise performance is positive. The paper clarifies both the impact of artificial intelligence on the energy efficiency of manufacturing enterprises and its mechanism of action; this will help provide a reference for future decision-making designed to improve manufacturing enterprises’ energy efficiency.

## 1. Introduction

For some time, the global energy issue has been a major concern, hindering the development of human society [1,2]. The 2019 BP World Energy Statistical Yearbook shows that, in 2018, global primary energy demand increased 2.9% and carbon emissions increased 2.0%. This was the fastest growth year since 2010. In 2019, affected by the new coronavirus epidemic, the growth rate of global primary energy consumption slowed to 1.3% as compared to 2018, but carbon emissions caused by energy consumption increased significantly, by 2.0% [3]. China accounts for more than three-quarters of the net increase in global energy consumption and has become its largest driving force. For the sustainable development of both the economy and society, the Chinese government has put energy conservation and emissions reduction front and center [4]. At the 2021 China Energy Work Conference, there was a call for the strict implementation of a “dual control” system involving both total energy consumption and intensity, with total energy consumption to be limited to within five billion tons of standard coal at an average annual growth rate of less than 3%.

The industrial sector is the largest consumer of energy. According to data from the United Nations Industrial Development Organization, in developing countries and countries with economies in transition, the growth rate of industrial energy use will be 1.8–3.1% per year, with 50% of energy to be supplied to industrial systems. At the same time, the contradiction between economic development and limited energy supply has become increasingly prominent. Therefore, how to manage the energy demand of Chinese manufacturing enterprises and improve their energy efficiency is very important for achieving regional and global reductions in greenhouse gas emissions and reducing corporate energy intensity [5]. Studies to date have pointed out that technology can indeed improve energy efficiency and reduce energy consumption [6,7], but current industrial energy efficiency is far below the best technically feasible levels.

**Figure 1:**
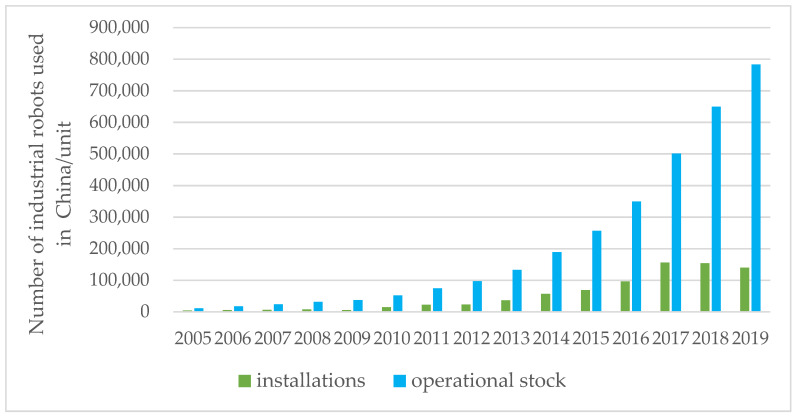
Installation and use of industrial robots in China.

With the rise of a new global scientific and technological revolution, AI has developed rapidly around the world and has now become an important developmental trend in global manufacturing. The use of industrial robots is an important manifestation of the application of AI in the manufacturing sector [8,9]. From the perspective of the application of industrial robots in China (see Figure 1), although the number of industrial robots put into use is increasing year by year, its growth rate is far lower than that for the number of industrial robots purchased in China. That is, companies have purchased artificial intelligence (AI) equipment, but the proportion of production applications is not high. There is a practical problem: the operation of artificial intelligence requires a lot of energy. After it is put into production, can artificial intelligence improve manufacturing enterprises’ energy efficiency? Manufacturing enterprises may have more stringent technical conditions for using AI and may face higher investment costs, resulting in the number of purchases of AI being greater than the number of applications for their use. Will this lead to a waste of resources for the company, thereby inhibiting energy efficiency? The questions to be studied in this paper are as follows:

- What impact does artificial intelligence have on the energy efficiency of Chinese manufacturing enterprises? How specific is the impact?
- In what ways does artificial intelligence affect the energy efficiency of manufacturing enterprises?
- What kind of heterogeneity is there in the impact of artificial intelligence on the energy efficiency of manufacturing enterprises?

To answer the above questions, it is necessary to conduct empirical research on the basis of looking at related theories and combining them with real world data in China.

Most of the existing research on artificial intelligence and industrial robots has focused on the labor market, economic growth, carbon emissions, etc. [10,11,12]. However, empirical research between AI and energy efficiency is relatively rare. We aim to supplement this research herein. The purpose of this paper is to analyze the impact mechanism of artificial intelligence on the energy efficiency of manufacturing enterprises and to answer the question of how artificial intelligence affects energy efficiency. This paper uses the data of manufacturing enterprises to construct a DEA-Malmquist model and multiple fixed effect model for empirical testing. We further analyze the role of firm age and firm performance in moderating the impact of AI on energy efficiency. This paper provides micro-evidence for the impact of artificial intelligence on energy efficiency, and expands the research on artificial intelligence and enterprise energy efficiency.

The structure of this article is as follows: This first part reviews the related research on the factors affecting energy efficiency. Through the method of literature review, it analyzes how artificial intelligence has an impact on energy efficiency and proposes research hypotheses. The second part is the literature review and research hypothesis. The third part is model design, and mainly deals with model design, variable selection and descriptive statistics of data. The main goal is to build an econometric model that empirically tests the impact of AI on energy efficiency. A data envelopment model is established to measure the energy efficiency of manufacturing enterprises. Finally, select the relevant control variables and describe the data. The fourth part is the empirical test. This part mainly analyzes the empirical results and uses the instrumental variable method (IV) to alleviate the endogeneity problem of the model and to discuss the heterogeneity of enterprises. The fifth part is discussion. This part will use the literature comparison method to compare the conclusions of this paper with the published literature, answer the research questions of this paper and summarize its contributions. The last part is the conclusion and policy recommendations.

## 2. Literature Review and Research Hypothesis

With the increasingly prominent energy problem, discovering the factors that affect energy efficiency has become the focus of scholars’ research, including urbanization level [13], energy cost [14], environmental regulation [15,16], and resource endowment [17], etc.

Relevant research on the impact of technological progress on corporate energy efficiency can be divided into two categories. One view is that technological advancement can improve corporate energy efficiency and thereby reduce energy consumption [18,19,20] Popp [21] used patent data to estimate the impact of technological progress on energy consumption. The results of the study proved that technological advancement can save enterprises more energy in the long run. Welsch and Ochsen [22] demonstrated, through an empirical study in the Federal Republic of Germany, that technological progress can improve corporate energy efficiency, while factor substitution and biased technological progress are important factors for fluctuations in energy intensity. Technological progress can effectively narrow the energy efficiency gap between European companies. In the future, the EU should support European manufacturing companies in introducing and using both sustainable processes and product innovation to narrow the energy efficiency gap [23]. Wang and Wang [24], using the number of patents granted to measure technological innovation, found that technological innovation in China has significantly improved urban energy efficiency. Sun et al. [25], based on their research on 24 innovative countries, found that there is a significant positive relationship between technological innovation and energy efficiency.

Other scholars believe that innovations in artificial intelligence, information and communication technology have led to a decrease in the unit cost of energy. This will stimulate enterprises to expand production, bring on a “rebound effect” to energy consumption and thus lead to a more complex kind of energy efficiency for manufacturing enterprises [26,27]. Currently, academia has widely accepted the existence of the rebound effect [28,29,30], although it is still controversial as to whether the rebound effect will completely offset increases in energy efficiency brought about by technological progress (that is, the rebound effect is greater than 100%). A study by Jin [31], based on the electricity consumption data of 3500 households in South Korea, gave empirical calculations showing that the energy rebound effect was about 30%. Vélez-Henao et al. [32] believed that every 1% drop in energy prices in Colombia would increase the rebound effect by 38.56%. Adha et al. [33] found that the short-term and long-term rebound effects in Indonesia were 87.2% and −45.5%, respectively, indicating that technological improvements can improve energy efficiency in the long-term. Therefore, technological progress has become an important factor affecting energy efficiency.

Artificial intelligence is considered to be a general technology that can support other innovations [34]. While enterprises use AI to achieve technological progress, the use of AI further promotes new technological innovations, thus forming a new virtuous circle [35]. Therefore, considering that related research on the impact of AI on corporate energy efficiency is still relatively rare, we look first at research showing how technological progress impacts corporate energy efficiency. This will significantly affect energy efficiency [34].

First, artificial intelligence can accelerate knowledge spillover and creation and promote technological progress of enterprises in energy saving and cleaner production, and thereby improve energy efficiency [25]. The stronger the ability of an enterprise to learn and absorb, the higher its ability to innovate [36]. Through deep learning and computer vision technology, artificial intelligence can screen out a large amount of effective information and create new knowledge and new computing solutions more efficiently than ever before, thereby accelerating the process of knowledge reorganization [37]. The acceleration of knowledge reorganization can promote the re-creation of knowledge and information [38]. At the same time, artificial intelligence breaks the boundaries of knowledge dissemination within and between enterprises and can accelerate knowledge spillover and information sharing, thereby promoting technological innovation [39]. With the improvement in the level of artificial intelligence, this information contribution ability has been further strengthened. The learning and absorptive capacity of the employees of the enterprise is also continuously improved, thereby promoting the absorption and creation of knowledge within the enterprise [34]. In turn, this promotes technological innovation of enterprises, results in more optimized equipment and energy use decisions, and improves energy efficiency [35].

Second, artificial intelligence promotes technological progress and improves energy efficiency by increasing investment in R&D and talent. The development of artificial intelligence will bring more intelligent devices such as industrial robots, thereby producing a labor substitution effect [40]. The shortage of high-skilled labor caused by this complementary substitution of labor will further force manufacturing enterprises to increase investment in talents and R&D [41]. Talent and R&D investment can further promote technological progress [42]. At the same time, with the increasing global trend of using industrial robots, companies are actively improving their production processes and manufacturing skills. Among them, the use of artificial intelligence technology to improve product processes has become an important way to gain competitive advantage [43]. In turn, through technological progress, the production process is optimized, thereby improving energy efficiency [44]. In the waste management sector, the application of neural networks and machine learning can predict the amount of waste generated, promote waste reuse and improve energy efficiency [45].

In addition, artificial intelligence can improve energy efficiency by increasing technological efficiency. That is, AI can also shorten the gap between businesses and optimal energy efficiency by improving technological efficiency. On the one hand, artificial intelligence can improve production efficiency. Manufacturing companies can use industrial robots to replace low-skilled production workers [46]. Using intelligent technology for production can effectively improve product quality and reduce energy consumption caused by repeated production due to substandard products [47]. With the help of artificial intelligence technology, such as machine learning, deep learning, etc., enterprises can complete the design, production and sales of products faster [48,49]. On the other hand, artificial intelligence can improve the efficiency of resource allocation. Enterprises use advanced intelligent equipment to make equipment self-perceive, self-analyze, and self-decide. This results in real-time feedback and optimization of production information, reduces equipment response time, reduces energy waste and significantly improves resource allocation efficiency and energy efficiency [50,51].

Artificial intelligence has been widely used in various sectors to improve energy efficiency [52,53]. For example, in the construction sector, the combination of artificial intelligence and big data can improve the energy efficiency of buildings and the comfort of houses [54]. In the energy supply sector, the application of smart meter data can help to accurately predict the consumption of electricity and natural gas so as to better plan and operate the energy supply system [55,56]. Huang and Koroteev [45] believe that AI technologies such as neural networks and machine learning are more successful in energy and waste management, which can then be used to improve the efficiency of electricity, heat and gas in the future. Chen et al. [57] believe that artificial intelligence can optimize equipment scheduling and operation, and their proposed AIEM model can effectively improve energy efficiency and promote the use of renewable energy.

Based on the above analysis, we propose the following hypothesis:

**Hypothesis** **1:** *AI can improve the energy efficiency of manufacturing companies.*

**Hypothesis** **2:** *AI can improve corporate energy efficiency by promoting technological progress and technical efficiency.*

## 3. Model Design

### 3.1. The Model

Referring to the research of Bloom et al. [58], we take total factor productivity (TFP) as the explained variable and establish the following regression equation:

(1) $$TFP_{ijct}=\alpha +\beta AI_{ijct}+\gamma {X^{\prime }}_{ijct}+\varphi_{ind}+\phi_{year}+\theta_{city}+\delta_{ijct}$$

where $TFP_{ijct}$ represents the total factor productivity of manufacturing enterprises in industry j in city i in year t, $AI_{ijct}$ represents the intelligence level of manufacturing enterprises and ${X^{\prime }}_{ijct}$ represents other control variables in the model that affect the total factor energy efficiency of manufacturing enterprises. $\varphi_{ind}$, $\phi_{year}$ and $\theta_{city}$ represent industry fixed effects, time fixed effects and city fixed effects, respectively. $\delta_{ijct}$ is the random error term. β is the most concerned coefficient of this article. If β is statistically significantly positive, it means that the application of artificial intelligence has improved energy efficiency.

### 3.2. The Variables

(1) Dependent variable: total factor energy efficiency (TFP).

This paper refers to the research of Wang et al. [59] and calculates the total factor productivity of manufacturing enterprises based on the DEA-Malmquist index method. In recent years, Data Envelopment Analysis (DEA) has been often used by scholars to measure total factor productivity. The DEA method uses linear optimization to estimate the boundary production function and distance function, without making assumptions about the form and distribution of the production function, and so avoids strong theoretical constraints [60,61]. At the same time, the DEA-Malmquist index method is now one of the mainstream DEA measurement methods. It can decompose changes in total factor productivity into technological progress and changes in technical efficiency, thereby facilitating in-depth analysis of the causes of changes in total factor productivity. It has been widely used by scholars in energy efficiency research [62]. In this light and, referring to the research of Wang et al. [59], we assume that the form of the production function of the firm is in the form of the Cobb–Douglas production function, and its natural logarithm can be converted to a linear form:

(2) $$\ln(Y_{jt})=A+a\;ln\left(K_{jt}\right)+b\ln(L_{jt})+c\;ln(E_{jt})+\varepsilon_{jt}$$

where j and t represent the manufacturing enterprises and year, respectively, and $Y_{jt}$ represents the output, which is measured by the company’s operating income. $K_{jt}$, $L_{jt}$ and $E_{jt}$ represent the three production input factors of capital, labor and energy, respectively, which are measured by the total assets of the enterprise, the number of employees and the electricity consumption. A is a constant term, $\varepsilon_{jt}$ is a residual term and a, b and c are the coefficients of the elements. Therefore, by constructing the DEA- Malmquist model, we can calculate the total factor productivity (TFP) of manufacturing enterprises. According to the research of Fare et al., under the conditions of fixed returns to scale (*c*) and strong disposal of factors, the minimum technical efficiency (CRS) can be decomposed into:

(3) $$F_{j}^{t}(y^{t},x^{t}|c,s=s_{j}^{t}(y^{t},x^{t}|s)\cdot CN_{j}^{t}(y^{t},x^{t}|v)\cdot F_{j}^{t}(y^{t},x^{t}|v,w)$$

where $F_{j}^{t}(y^{t},x^{t}|c,s$ is the technical efficiency, $s_{j}^{t}\left(y^{t},x^{t}\vee s\right)$ is the scale efficiency, $CN_{j}^{t}\left(y^{t},x^{t}\vee v\right)$ is the degree of strong disposal of the measuring element and $F_{j}^{t}\left(y^{t},x^{t}\vee v,w\right)$ is the pure technical efficiency. The input distance function is the reciprocal of technical efficiency, namely:

(4) $$D_{j}^{t}\left(y^{t},x^{t}\right)=\frac{1}{F_{j}^{t}(y^{t},\;x^{t}|c,s)}$$

In Equation (4), the input distance function can be regarded as the distance moved from a certain production point $\left(y^{t},x^{t}\right)$ to the ideal input point, $D_{j}^{t}\left(y^{t},x^{t}\right)\geq 1$, when $D_{j}^{t}\left(y^{t},x^{t}\right)=1$, $\left(y^{t},x^{t}\right)$ is on the best front and the technology is valid; if $D_{j}^{t}\left(y^{t},x^{t}\right)>1$, then $\left(y^{t},x^{t}\right)$ is outside the best front and the technology is invalid.

Therefore, the Malmquist indices based on periods t and t+1 are:

$$M_{j}^{t}=\frac{D_{j}^{t}\left(x^{t+1},y^{t+1}\right)}{D_{j}^{t}\left(x^{t},y^{t}\right)}$$

(5) $$M_{j}^{t+1}=\frac{D_{j}^{t+1}\left(x^{t+1},y^{t+1}\right)}{D_{j}^{t+1}\left(x^{t},y^{t}\right)}$$

The above indexes are symmetrical in economic meaning. Referring to the method of Fare et al. (1994) [63], their geometric average is defined as a composite index, that is, total factor productivity TFP:

(6) $$TFP_{jt}=M_{j}\left(x^{t},y^{t},x^{t+1},y^{t+1}\right)={\left(M_{j}^{t}\cdot M_{j}^{t+1}\right)}^{\frac{1}{2}}={\left[\frac{D_{j}^{t}\left(x^{t+1},y^{t+1}\right)}{D_{j}^{t}\left(x^{t},y^{t}\right)}\cdot \frac{D_{j}^{t+1}\left(x^{t+1},y^{t+1}\right)}{D_{j}^{t+1}\left(x^{t},y^{t}\right)}\right]}^{\frac{1}{2}}$$

We further decompose the Malmquist index into:

(7) $$TFP_{jt}=M_{j}\left(x^{t},y^{t},x^{t+1},y^{t+1}\right)=\frac{D_{j}^{t+1}\left(x^{t+1},y^{t+1}\right)}{D_{j}^{t+1}\left(x^{t},y^{t}\right)}\times {\left[\frac{D_{j}^{t}\left(x^{t},y^{t}\right)}{D_{j}^{t+1}\left(x^{t},y^{t}\right)}\cdot \frac{D_{j}^{t}\left(x^{t+1},y^{t+1}\right)}{D_{j}^{t+1}\left(x^{t+1},y^{t+1}\right)}\right]}^{\frac{1}{2}}\times \;{\left[\frac{D_{j}^{t}(x^{t+1},y^{t+1})/D_{j}^{t}\left(x^{t+1},y^{t+1}\right)}{D_{j}^{t}(x^{t},y^{t})/D_{j}^{t}\left(x^{t},y^{t}\right)}\cdot \frac{D_{j}^{t+1}(x^{t+1},y^{t+1})/D_{j}^{t+1}\left(x^{t+1},y^{t+1}\right)}{D_{j}^{t+1}(x^{t},y^{t})/D_{j}^{t+1}\left(x^{t},y^{t}\right)}\right]}^{\frac{1}{2}}=pech\times techch\times \;sech$$

where pech is pure technical efficiency change, which is the change in technical efficiency under the assumption of variable returns to scale. sech is the change in scale efficiency, indicating the influence of scale economy on total factor energy efficiency. pech×sech is the change in technical efficiency (eff), which measures the degree of catching up to the best practice of each observation object from t to t+1. Greater than 1 means that the technical efficiency is improved, less than 1 means that the technical efficiency is reduced and equal to 1 means that there is no change in the technical efficiency.

Techch is the change in technological progress and reflects the contribution of the movement of the production front to the change in total factor productivity. It measures the movement of the technological boundary from t to the t+1 period. Greater than 1 means technological progress, less than 1 means technological regression and equal to 1 means no change in technological level.

(2) Independent variable: Enterprise intelligence level (AI).

This article refers to the research of Acemoglu and Restrepo [40] and uses the number of industrial robots to measure the level of artificial intelligence of manufacturing enterprises. With reference to the research of Yang and Hou [64], we calculate the use of industrial robots in manufacturing enterprises based on the ratio of the output value of the enterprise to the total output value of the industry to measure the level of enterprise intelligence.

(3) Control variables: The debt-to-asset ratio (Lcv).

The debt-to-asset ratio measures the ability of manufacturing enterprises to use creditors to provide funds for operating activities. Companies with high levels of debt lack sufficient capital to use advanced technology and optimize production processes; it is difficult to improve their energy efficiency. This article uses the ratio of the total amount of corporate liabilities to total assets to measure debt-to-asset ratio.

Corporate age (Firmage). Generally speaking, the rigidity of the corporate structure, caused by the age of manufacturing enterprises, will affect the company’s energy structure adjustment, thereby affecting the energy efficiency of manufacturing enterprises. We take the company’s incorporation date as the benchmark and add the company’s age variable to the model.

Ownership of enterprises (Ownership). Generally speaking, private enterprises are more likely to take measures to reduce the cost of production and operation, which will affect the total factor energy efficiency of manufacturing enterprises. If the company is a private company, it is 1, and for the rest it is 0.

Corporate performance (Ros). Corporate performance often has positive and negative effects on total factor energy efficiency. When the company’s performance is good, the company may choose to expand the scale of production and invest in more factor resources. The positive effect lies in the scale economy effect and output growth brought about by the expansion of production scale, which leads to the growth of the company’s total factor energy efficiency. The negative effect is when the marginal increase in output is smaller than the increase in input energy, which leads to a reduction in total factor energy efficiency. Referring to the study of Boubakri et al. [65], we use the ratio of corporate net profit to operating income to measure ros.

Enterprise energy consumption level (Energy). Differences in enterprise energy consumption levels will lead to changes in total factor energy efficiency. Manufacturing enterprises with higher levels of energy consumption may have greater marginal room for growth in their total factor energy efficiency. At the same time, energy dependence may also lead to smaller changes in their total factor energy efficiency. According to the “2010 National Economic and Social Development Statistical Report”, companies in the six highest energy consumption industries specified by the state are assigned a value of 1, and the rest are assigned a value of 0.

### 3.3. Data Sources

The data of industrial robots in this article come from the International Federation of Robotics (IFR), which counts the global number of industrial robots by industry. The enterprise-level data come from a comprehensive survey conducted by the Guangdong Provincial Economic and Information Technology Commission on the situation of enterprises above a designated size in the province. The data span from 2013 to 2015. As the database counts more than 110,000 manufacturing enterprises in Guangdong Province, the data are considered comprehensive and can be used to scientifically measure the energy utilization of micro-enterprises.

On this basis, this article processes the data as follows: (1) The World Robot Association data are first sorted according to the “Classification of National Economic Industries” (2019). (2) Non-manufacturing industry data and samples of manufacturing enterprises with less than ten employees are excluded, following which abnormal values of various variables are processed. (3) In order to further alleviate the problem of heteroscedasticity caused by variable measurement, this paper performs winsorized processing for both dependent variables and independent variables below the 1% quantile and above the 99% quantile. The explanation and data sources of variables are listed in Table 1. The descriptive statistics of the processed variables are shown in Table 2.

## 4. Empirical Test

### 4.1. Benchmark Regression

In order to exclude the influence of individual manufacturing enterprises’ characteristics on the robustness of the model, we use a two-way fixed effects model for empirical testing. Compared with the general static panel model that only fixes individual corporate effects that does not change with time, the two-way fixed effects model fixes the individual corporate effects and time effects, respectively; this makes the empirical results more credible. The benchmark regression results are shown in Table 3.

Model (1) only adds independent variables. The results show that the coefficient of the Ai variable is 0.0469 and that it passes the 1% significance test. Model (2) controls the fixed effects of industry, region and time on the basis of Model (1). The results also show that artificial intelligence is positively correlated with energy efficiency of manufacturing enterprises. The coefficient of artificial intelligence is 0.1450 and passes the 1% significance test. In Model (3), we add control variables; the results show that the coefficient of artificial intelligence is 0.0458, and that it passes the 1% significance test. Model (4) controls the fixed effects of industry, region and time on the basis of Model (3). The results show that the artificial intelligence coefficient is 0.1449, and that it passes the 1% significance test, further proving that artificial intelligence has a significant positive correlation with manufacturing companies. From this, we deduce that artificial intelligence has a significant positive impact on the energy efficiency of manufacturing enterprises, which is consistent with Hypothesis 1. Artificial intelligence can improve energy efficiency by improving both the production efficiency and the management efficiency of manufacturing enterprises.

### 4.2. Endogenous Test

There may be an endogenous problem in Equation (1) in this paper. First, there is the problem of missing unobservable variables, such as production and operation problems that may affect both the artificial intelligence level and the energy efficiency of manufacturing enterprises at the same time. The second is that manufacturing enterprises with higher energy efficiency in production may often be manufacturing enterprises with higher levels of artificial intelligence, so there are synergy biases. Therefore, this article alleviates the endogenous problem by looking for an instrumental variable method. The Acemoglu and Restrepo [40] study pointed out that, due to the obvious international competition among several major manufacturing countries in the world, countries have shown a high degree of convergence in the scale of new technologies and equipment applications. Therefore, it is reasonable to use the number of industrial robots in the same industry in other major manufacturing countries as an instrumental variable.

Considering the specific situation of China’s manufacturing industry, the competition between China’s manufacturing industry and the United States has become increasingly fierce in recent years, with the manufacturing industries of the two countries having a strong competitive relationship. At the same time, data from the International Federation of Robotics (IFR) show that the number of industrial robots used in China and the United States is also increasing sharply, with a strong positive correlation. Therefore, this article draws on the ideas of Acemoglu and Restrepo [40] and uses the number of industrial robots in the same industry in the United States during the same period as the instrumental variable iv, which conforms to the correlation assumption of instrumental variables. On the other hand, the use of artificial intelligence in the United States has a relatively small impact on the energy efficiency of manufacturing enterprises in Guangdong Province, China, which conforms to the exogenous hypothesis of instrumental variables. The test results are shown in Table 4.

This paper uses the two-stage least squares method to estimate Equation (1). The one-stage regression results in the Model (5) in Table 3 show that the instrumental variables selected in this paper are significantly positively correlated with the endogenous variables. The correlation coefficient is 0.0185, which passes the 1% significance test and satisfies the correlation hypothesis. The two-stage regression result in Model (6) shows that the sign of the coefficient of artificial intelligence is positive, the coefficient is 0.8875 and that it is significant at the 5% level, which is consistent with the benchmark regression result and further proves Hypothesis 1. At the same time, this article further tests the rationality of the instrumental variables and rejects the null hypothesis, which proves that the instrumental variables used in this article are appropriate.

### 4.3. Robustness Test

In order to further prove the robustness of the model, we conducted a robustness test as shown in Table 5.

The first step is to replace the explanatory variables. In Model (7), we take the industrial robot inventory (Ai2) of manufacturing enterprises as a substitute independent variable to measure the level of artificial intelligence. The estimation results show that the inventory coefficient of industrial robots is 0.0511 and that it passes the 1% significance test, indicating that there is a significant positive correlation between industrial robot inventory and energy efficiency of manufacturing enterprises. This result is consistent with the benchmark regression results and proves that artificial intelligence has a significant positive impact on the energy efficiency of manufacturing enterprises.

The second is to replace the explained variable. In Model (8), referring to the study of Bloom et al. [58], energy intensity (ee) is used as a substitute dependent variable. Energy intensity reflects the energy consumption of a company’s unit output value and can measure the energy efficiency of a company to a certain extent. The results show that the coefficient of influence of artificial intelligence on the energy intensity variables of manufacturing enterprises is −0.0053, which passes the 1% significance test. It shows that artificial intelligence has significantly reduced the energy intensity of manufacturing enterprises and that manufacturing enterprises have enjoyed improved economic energy benefits. This proves that artificial intelligence can improve the energy efficiency of manufacturing enterprises.

The third step is to replace the regression model. In Model (9), taking into account the possible censorship features in the dependent variable, we use the Tobit model for regression. Under the Tobit regression model, the coefficient of influence of artificial intelligence on enterprise energy efficiency variables is 0.2991 and passes the 1% significance test. It shows that there is still a significant positive correlation between artificial intelligence and enterprise energy efficiency variables. The conclusions obtained are consistent with the OLS regression results, which proves that artificial intelligence can significantly promote the energy efficiency of manufacturing enterprises. In addition, we use the Sys-GMM method for regression in Model (10). The Sys-GMM method can further alleviate the endogenous problems that may exist in the model to a certain extent [66]. Under the Sys-GMM model, the impact of artificial intelligence on energy efficiency variables is still significantly positive.

### 4.4. Heterogeneity Test

Considering that the level of artificial intelligence of manufacturing enterprises will be affected by the characteristics of individual enterprises, we expand Equation (1) to increase the interaction term between the individual heterogeneity characteristic variable ($char_{ijct}$) of manufacturing enterprises and artificial intelligence (AI). To test the effect of artificial intelligence on the energy efficiency of manufacturing enterprises under conditions of differing individual enterprise heterogeneity, the expanded equation is as follows:

(8) $$TFP_{ijpt}=\alpha +\beta AI_{ijpt}+\gamma {X^{\prime }}_{ijpt}+\tau rob_{ijpt}*char_{ijct}+\varphi_{ind}+\phi_{year}+\theta_{city}+\delta_{ijpt}$$

For the characteristics of individual heterogeneity variables, this paper examines the two dimensions of firm age (firmage) and firm performance (ros), and the measurement method is the same as that of Equation (1). Regression results are shown in Table 6.

**Figure 2:**
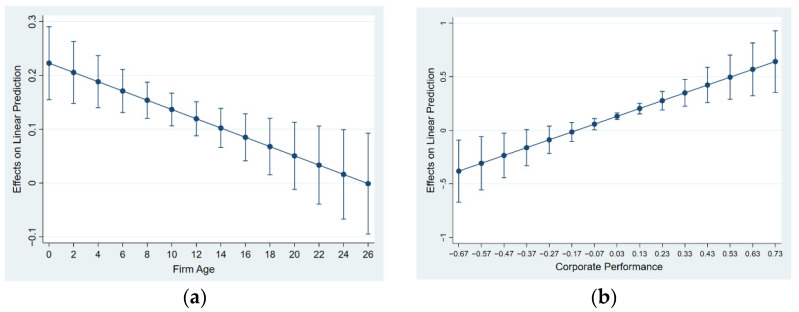
The average marginal effect of enterprise individual characteristic variables.

According to Model (11) in Table 6, the artificial intelligence coefficient is 0.2473 and passes the 1% significance test, indicating that artificial intelligence indeed has a significant positive impact on energy efficiency. The coefficient of the interaction term between enterprise age and artificial intelligence is significantly negative, showing that the age of the company will weaken the role of artificial intelligence in promoting energy efficiency in manufacturing enterprises. That is, the longer the enterprise has been established, the smaller the effect of artificial intelligence on its energy efficiency. According to the analysis of the inter-effect on the left side of Figure 2a, when the firm age does not exceed 18 years, the marginal effect of artificial intelligence on the energy efficiency of manufacturing enterprises gradually decreases and is statistically significant. When the age of manufacturing enterprise (firmage) exceeds 18 years, the marginal effect of artificial intelligence on energy efficiency gradually decreases and is not statistically significant. At the same time, according to Model (12) in Table 6, the artificial intelligence coefficient is 0.1172 and passes the 1% significance test. This also supports the results of the benchmark regression. The coefficient of the interaction term (AI×ros) between corporate performance and artificial intelligence is significantly positive, indicating that the performance of manufacturing enterprises can strengthen the role of artificial intelligence in promoting energy efficiency. That is, the higher the performance of manufacturing enterprises, the greater the effect of artificial intelligence in improving its energy efficiency. According to the analysis on the right side of Figure 2b, the marginal effect of artificial intelligence on the energy efficiency of manufacturing enterprises is negative and statistically significant when the enterprise performance term (ros) does not exceed −0.57. When the performance of manufacturing enterprise (ros) does not exceed −0.17, the marginal effect of artificial intelligence on the energy efficiency of manufacturing companies turns from negative to positive, but it is not statistically significant. When the company’s age exceeds −0.07, the marginal effect of artificial intelligence on the energy efficiency of manufacturing companies gradually increases and is statistically significant.

### 4.5. Influence Mechanism Test

Impact mechanism analysis shows that artificial intelligence can promote the improvement of energy efficiency by promoting technological progress and improving technical efficiency. Therefore, based on the Malmquist theory, we take the two decomposition indexes of technological progress (techch) and technical efficiency (eff) as dependent variables, and observe the different effects of intelligence on the two. The results are shown in Table 7, where Model (13) represents technological progress and Model (14) represents technical efficiency. According to the results, the influence coefficient of artificial intelligence on the technological progress variable is 0.1419, and it has passed the 1% significance test. This is consistent with Hypothesis 2. It shows that artificial intelligence can significantly promote the technological progress of manufacturing enterprises. However, the relationship between AI and technological efficiency failed the significance test. It shows that the impact of artificial intelligence on the technical efficiency of manufacturing enterprises is not obvious, which is different from Hypothesis 2.

## 5. Discussion

The application of AI technologies has had a significant impact on the energy sector [67]. The empirical evidence in this paper shows that artificial intelligence can significantly improve the energy efficiency of manufacturing enterprises. This conclusion is similar to that of Chen et al. (2021). Chen et al. (2021) proposed a new algorithm based on artificial intelligence technology and an evaluation model using AIEM for energy efficiency and conservation prediction. It is concluded that the use of artificial intelligence technology can improve energy efficiency and renewable energy use [57]. Furthermore, based on algorithms, Lee et al. (2022) concluded that artificial intelligence can achieve energy savings in different fields [68]. In contrast, we are not an algorithm or forecasting study, but based on econometrics, using micro-data of manufacturing companies, we have proved the impact of AI on energy efficiency. To the best of our knowledge, the paper is one of the few to provide evidence of the impact of AI on energy efficiency through econometric methods.

We believe that the promotion of artificial intelligence in energy efficiency mainly comes from two aspects. First, as a general-purpose technology, artificial intelligence can accelerate knowledge learning and creation, increase the R&D and talent investment of manufacturing enterprises and promote technological progress of manufacturing enterprises, thereby improving energy efficiency. This was also verified in the mechanism inspection. This conclusion is similar to the research conclusion of Fisher-Vanden et al. [69]. Technological progress has a significant effect on the improvement of energy efficiency in Chinese manufacturing enterprises. Artificial intelligence can significantly improve the progress of enterprise energy utilization technology by accelerating both enterprise knowledge learning and creation and increasing enterprise R&D and talent investment.

Second, artificial intelligence promotes energy efficiency by improving technical efficiency. However, this has not been verified in the mechanism test. We believe that, on the one hand, this may be due to the existence of the productivity paradox. Generally speaking, technological progress can effectively promote improvements in technical efficiency, but when it comes to computer-related technology, this experience is often proved to be wrong. The excessive automation brought about by artificial intelligence may restrict the growth of technical efficiency [40]. Excessive intelligence will not only directly lead to the reduction of technical efficiency, but may also cause an energy “rebound effect” through wastage of resources and labor mismatch and, in this way, indirectly inhibit the growth of energy efficiency. Judging from the current status of corporate energy efficiency, the impact of artificial intelligence on corporate energy efficiency is more from technological progress. The level of technical efficiency in various industries is still relatively average, and the growth rate is relatively slow. As predicted by Li and Zhou [70], as the market gradually improves, technological progress will continue to play a greater role in the energy efficiency growth of manufacturing companies, while the contribution of technical efficiency will be relatively reduced.

The test results of the heterogeneity of individual characteristics of manufacturing enterprises show that: On the one hand, the role of artificial intelligence in promoting energy efficiency will decrease as companies age. The reason may be that the older the enterprise, the higher the cost of coordinating various business units. Disadvantages such as high transformation costs and rigid corporate structure brought about by the age of manufacturing enterprises often inhibit the improvement of energy efficiency in manufacturing enterprises. On the other hand, the role of artificial intelligence in promoting energy efficiency will increase with the growth of corporate performance. This shows that an improvement in corporate performance is conducive to the rational operation of artificial intelligence companies. The application of artificial intelligence requires continuous capital investment by manufacturing enterprises. The better the performance of the enterprise, the more sufficient funds the manufacturing enterprises have to play the role of intelligent transformation. The study by Huang et al. (2022) also came to a similar conclusion. It is believed that the use of industrial robots will improve corporate performance, expand production scale and then improve energy efficiency through scale effects [71]. The previous literature, although analyzing the industry heterogeneity of the impact of industrial robots on energy intensity, believed that industrial robots mainly affect the energy intensity of labor-intensive industries. However, it is not specific to the level of firm heterogeneity [72].

Based on the above analysis and comparison with existing literature, the marginal contribution of this paper is as follows: (1) From the perspective of AI promoting technological progress and improving technical efficiency, this paper analyzes the impact mechanism of AI on the energy efficiency of manufacturing enterprises. (2) It constructs a DEA-Malmquist model to measure the total factor energy efficiency of micro-enterprises and empirically tests the impact of AI on the total factor energy efficiency of manufacturing enterprises and its heterogeneity. (3) To alleviate the endogenous problems in the model as much as possible, the number of industrial robots in the same industry in the United States during the same period is taken as an instrumental variable for the use of AI in Chinese manufacturing enterprises. (4) Using the data of manufacturing enterprises, this paper provides the first microscopic evidence that AI can improve the energy efficiency of manufacturing enterprises. It thus expands current research on the relationship between AI and manufacturing enterprises’ energy efficiency.

## 6. Conclusions 

This paper studies the impact of artificial intelligence on the energy efficiency of manufacturing enterprises and its mechanism of action from both theoretical and empirical aspects. Research shows that: Artificial intelligence significantly improves the energy efficiency of manufacturing companies. After introducing the US industrial robot data as an instrumental variable for endogeneity testing, the results are still stable.

In addition, we found that the impact of artificial intelligence on the energy efficiency of manufacturing enterprises is mainly achieved by accelerating knowledge learning and creation, increasing the R&D and talent investment of manufacturing enterprises and promoting the technological progress of manufacturing enterprises. This paper further analyzes the heterogeneity of manufacturing enterprises. The results show that the age of manufacturing firms inhibits the promotion of artificial intelligence on energy efficiency. Manufacturing firm performance enhances AI’s boost to energy efficiency, but this boost can only be seen when firm performance is positive.

Based on the above conclusions, the following policy recommendations are put forward:

(1) Increase the application scope of artificial intelligence in manufacturing enterprises and give full play to the positive impact of technological progress on enterprise energy efficiency. Encourage manufacturing enterprises to continuously increase investment in research and development of intelligent technology. Shift technological innovation from production efficiency-oriented to energy saving-oriented innovation.

(2) Improve the contribution of artificial intelligence to the technical efficiency of energy utilization. Through intelligent decision-making, intelligent control and intelligent management, manufacturing enterprises can allocate resources more reasonably in the process of production and operation, save energy costs, reduce energy waste and improve energy utilization efficiency.

(3) With artificial intelligence applications, focus on enterprise heterogeneity. Encourage high-tech manufacturing enterprises to vigorously develop artificial intelligence, give full play to the knowledge and technology spillovers brought by artificial intelligence, promote enterprise energy technology innovation and improve energy efficiency.

Due to data limitations, the data used in this article are the data of manufacturing enterprises from 2013 to 2015; the time span is relatively short. The conclusions drawn can only represent the short-term impact of artificial intelligence on the energy efficiency of manufacturing enterprises. The research conclusions in this paper are based on samples from Guangdong Province, China and may not be suitable for other countries. In addition, although this paper uses enterprise micro-survey data, there is still a lack of data to directly measure the level of artificial intelligence in enterprises. In the future, based on the global data, the long-term effects of artificial intelligence for manufacturing enterprises can be studied further.

## Figures and Tables

**Table 1:** Description of variables.

Variables	Symbol	Definition Measuring Method	Unit	Data Sources
Industrial robots	AI	Installation amount of industrial robots	1 unit	International Federation of Robotics (IFR)
Total factor energy efficiency	TFP	DEA-Malmquist model	/	The comprehensive survey conducted by the Guangdong Provincial Economic and Information Technology Commission on the situation of enterprises above a designated size in the province
Debt-to-asset ratio	Lcv	The ratio of the total amount of corporate liabilities to total assets	/	
Enterprise age	Firmage	The current date minus the enterprises’ registered date	year	
Ownership of enterprises	Owner-ship	If the enterprise is a private company, it is 1, and for the rest it is 0	/	
Enterprise performance	Ros	The ratio of net profit to operating revenue	/	
Enterprise energy consumption level	Energy	If enterprise is in the six high energy consumption industries, it is 1, and for the rest it is 0	/	

**Table 2:** Descriptive statistics of variables.

Variable	Obs	Std.Dev.	Mean	Min	Max
AI	40,053	0.3052	0.7087	0.0000	7.6321
Lcv	40,053	0.5480	0.3756	0.0000	2.9998
Firmage	40,053	8.1580	5.4697	0.0000	26.0000
Ros	40,053	0.0588	0.1409	−0.6713	0.7897
Ownership	40,053	0.9959	0.0641	0.0000	1.0000
Energy	40,053	0.1638	0.3701	0.0000	1.0000
TFP	40,053	1.1728	0.9552	0.0902	11.5830

**Table 3:** Benchmark regression.

Variable	(1)	(2)	(3)	(4)
	M1	M2	M3	M4
AI	0.0469 ***	0.1450 ***	0.0458 ***	0.1449 ***
	(5.99)	(8.70)	(5.79)	(8.74)
Control	NO	NO	YES	YES
Industry_FE	NO	YES	NO	YES
City_FE	NO	YES	NO	YES
Year_FE	NO	YES	NO	YES
cons	1.1585 ***	0.9974 ***	1.1898 ***	0.9708 ***
	(228.53)	(178.77)	(22.26)	(6.03)
N	40,053	40,053	40,053	40,053
${\mathrm{Adj}}_{R}^{2}$	0.0012	0.0234	0.0027	0.0246

Note a: (1) The values in parentheses are standard errors. (2) *** indicate that the variable coefficients have passed the 1% significance tests, respectively. Note b: N represents the number of sample observations.

**Table 4:** Endogenous test.

Variable	(5) First	(6) 2SLS
AI		0.8875 **
		(2.07)
IV	0.0185 ***	
	(7.92)	
Control	YES	YES
Industry_FE	YES	YES
City_FE	YES	YES
Year_FE	YES	YES
N	39854	39,854
Underidentificationtest		
Kleibergen–PaaprkLMstatistic	60.52 ***	
Weakidentificationtest		
Cragg–DonaldWaldFstatistic	14.48 ***	
Kleiberge–PaapWaldrkFstatistic	62.68 ***	
	(16.38)	
*Weakinstrumentrobustinference*		
*Anderson*–*RubinWaldtest*	4.54 **	

Note a: (1) The values in parentheses are standard errors. (2) ***, ** indicate that the variable coefficients have passed the 1% and 5% significance tests, respectively. Note b: The blanks indicate that the relevant variables are not included in the model.

**Table 5:** Robustness test.

Variable	(7)	(8)	(9)	(10)
	Replace Explanatory Variables	Replace Dependent Variable	Tobit	Sys-GMM
AI		−0.0053 ***	0.0143 ***	0.2991 ***
		(−3.80)	(4.30)	(7.84)
AI2	0.0511 ***			
	(8.00)			
L.tfee				−0.1094 ***
				(−6.83)
Control	YES	YES	YES	YES
Industry_FE	YES	YES	YES	YES
City_FE	YES	YES	YES	YES
Year_FE	YES	YES	YES	YES
_cons	0.9656 ***	0.0616 ***	3.4522	1.5660 ***
	(5.97)	(3.53)	(0.14)	(5.91)
N	40,053	40,053	40053	24,729
$\mathrm{Adj}\text{\_}R^{2}$	0.0243	0.0053		

Note a: (1) The values in parentheses are standard errors. (2) *** indicate that the variable coefficients have passed the 1% significance tests, respectively. Note b: The blanks indicate that the relevant variables are not included in the model.

**Table 6:** Heterogeneity test.

Variable	(11) Firmage	(12) Ros
AI	0.2473 ***	0.1172 ***
	(6.77)	(6.54) *
AI∗Firmage	−0.0107 ***	
	(−3.60)	
AI∗Ros		0.6229 ***
		(2.88)
Control	YES	YES
Industry_FE	YES	YES
City_FE	YES	YES
Year_FE	YES	YES
cons	0.9462 ***	0.9688 ***
	(5.87)	(6.00)
N	40053	40053
${\mathrm{Adj}}_{R}^{2}$	0.0252	0.0260

Note a: (1) The values in parentheses are standard errors. (2) ***, * indicate that the variable coefficients have passed the 1% and 10% significance tests, respectively. Note b: The blanks indicate that the relevant variables are not included in the model.

**Table 7:** Heterogeneity test.

Variable	(13) Techch	(14) Eff
AI	0.1419 ***	−0.0061
	(9.31)	(−1.53)
Control	YES	YES
${\mathrm{Industry}}_{\mathrm{FE}}$	YES	YES
${\mathrm{City}}_{\mathrm{FE}}$	YES	YES
${\mathrm{Year}}_{\mathrm{FE}}$	YES	YES
cons	0.9644 ***	1.0276 ***
	(6.38)	(23.43)
N	39785	38905
${\mathrm{Adj}}_{R}^{2}$	0.0128	0.0887

Note a: (1) The values in parentheses are standard errors. (2) *** indicate that the variable coefficients have passed the 1% significance tests, respectively. Note b: The blanks indicate that the relevant variables are not included in the model.

## Data Availability

The data of industrial robots in this article comes from the International Federation of Robotics (IFR). This data can be found here: https://ifr.org/worldrobotics/ (accessed on 12 December 2021). The enterprise-level data comes from a comprehensive survey conducted by the Guangdong Provincial Economic and Information Technology Commission on the situation of enterprises above a designated size in the province. The data are survey data and have not been made public.
